# Herbal Formula, PM014, Attenuates Lung Inflammation in a Murine Model of Chronic Obstructive Pulmonary Disease

**DOI:** 10.1155/2012/769830

**Published:** 2012-06-12

**Authors:** Hyojung Lee, Youngeun Kim, Hye Jin Kim, Soojin Park, Young Pyo Jang, Sungki Jung, Heejae Jung, Hyunsu Bae

**Affiliations:** ^1^Department of Oriental Physiology, College of Oriental Medicine, Kyung Hee University, Seoul 130-701, Republic of Korea; ^2^Division of Pharmacognosy, College of Pharmacy, Kyung Hee University, Seoul 130-701, Republic of Korea; ^3^Department of Life and Nanopharmaceutical Sciences, College of Pharmacy, Kyung Hee University, Seoul 130-701, Republic of Korea; ^4^Division of Allergy and Respiratory System, Department of Oriental Internal Medicine, College of Oriental Medicine, Kyung Hee University, Seoul 130-701, Republic of Korea

## Abstract

Chronic obstructive pulmonary disease (COPD), which is characterized by airway obstruction, leads to, as the two major forms of COPD, chronic bronchitis and emphysema. This study was conducted to evaluate the effects of herbal formula, PM014, in a murine model of COPD. Balb/c mice were treated once with each herb extract in PM014 or PM014 mixture via an oral injection. Lipopolysaccharide (LPS) or elastase/LPS were administrated to the mice to induce a disease that resembles COPD. PM014 treatment significantly attenuated the increased accumulation of immune cells in bronchoalveolar lavage fluid (BALF) compared to control mice. In addition, the TNF-**α** and IL-6 levels in BALF were decreased in the PM014 mice. Furthermore, histological analysis demonstrated that PM014 attenuated the hazardous effects of lung inflammation. These data suggest that PM014 exerts beneficial effects against forms of COPD such as lung inflammation.

## 1. Introduction

Chronic obstructive pulmonary disease (COPD) is a very common public health concern worldwide, and the incidence of this disease is increasing globally [1, 2]. COPD is characterized by progressive and irreversible airway obstruction [3]. Current conventional treatment is geared toward relieving symptoms, preventing recurrent exacerbation, preserving optimal lung function, and enhancing the overall quality of life [4]. Although many drugs are used to treat COPD, adverse effects associated with several classes of drugs, such as steroids, have increased the need for alternative treatments such as herbal medicines [5, 6].

Because COPD is a chronic inflammatory disorder, it is essential to determine if novel anti-inflammatory agents can halt or slow the decline in lung function that occurs in response to this disease when selecting candidate drugs. Several studies have demonstrated that compounds derived from plants have anti-inflammatory or immune-modulating properties [7], and several herbal medicines, including *Panax ginseng* and *Salvia miltiorrhiza*, have been used for the treatment of COPD [8]. 

Chung-Sang-Bo-Ha-Tang (CSBHT) has been used to treat chronic pulmonary disease in Korea for centuries. Recently, it is well reported that CSBHT can reduce ovalbumin-induced chronic lung inflammation [9]. However, CSBHT contains 18 species of medicinal plants, which makes it difficult to standardize herbal formular [9]. Therefore, CSBHT was modified to PM014, contains 7 species of medicinal plants, and was evaluated to determine if it had an effect on lung inflammation that prevented the progression of COPD. 

To accomplish this, a mouse model of LPS-induced lung neutrophilia was used to mimic a COPD-like acute disease [10]. LPS is an endotoxin produced by gram-negative bacteria that induces an influx of inflammatory cells such as macrophages, neutrophils, and lymphocytes, while increasing the release of proinflammatory cytokines [11, 12]. In addition, many studies have reported that repeated LPS exposure induces pathological changes similar to those observed in COPD patients such as goblet cell hyperplasia in the airways [13] and chronic neutrophilia in the bronchi [14]. Our results demonstrated that PM014 administration can prevent inflammatory processes in the lungs.

## 2. Materials and Methods

### 2.1. Reagents

Seven medical herbs were used to prepare PM014 (Table 1). All herb extracts were purchased from Sun Ten (Sun Ten Pharmaceutical Co., Ltd., Taiwan). Mixtures of PM014 and each herb extract were dissolved in 0.9% saline to give a final concentration of 10% (w/v). The herb suspension supernatant (HSS) was then obtained by centrifugation at 27,000 ×g for 20 min at 4°C. The HSS was then passed through a sterile 0.20 *μ*m pore size filter unit (Sartorius AG, Germany). LPS (Calbiochem, Germany) was dissolved in 0.9% saline at a concentration of 8 mg/mL. Dexamethasone (Sigma Aldrich, USA), which was used as a positive control, was also dissolved in saline.

### 2.2. DART-MS Analysis of PM104

DART-MS (Direct Analysis in Real Time-Mass Spectrometry) measurement of PM104 was conducted using an AccuTOF single-reflectron time-of-flight mass spectrometer (JEOL Ltd., Tokyo, Japan) equipped with a direct analysis in real time (DART) ion source (IonSense, Saugus, MA, USA). The parameters of the mass spectrometer were slightly modified from those previously reported [15]. Mass calibration was accomplished by placing polyethylene glycol (PEG) with an average molecular weight of 600 amu (PEG 600) onto a glass rod and putting it into the ion source for accurate mass measurements and elemental composition calculations. Using a 10 L glass capillary (Paul Marienfeld GmbH, Germany), 2-3 mg of powder was packed and then directly introduced into the ion source.

### 2.3. Animals

Balb/c male mice (6 wk of age, weighing 20 g–25 g) were purchased from Orient Bio Inc. (Korea). All mice were maintained under specific pathogen-free conditions during the experiments, which were performed according to the ethical principles and guidelines established by the Kyung Hee University for the care and use of experimental animals. 

### 2.4. Animal Treatment and Induction of Lung Inflammation

To compare the therapeutic efficacy of each herb and PM014, each group of mice was administered PM014 (300 mg/kg) or each herb extract (in proportion to PM014) via an oral injection 2 hr prior to LPS stimulation. The adequate dosage of PM014 was examined by an in vivo study in which various amounts were administered (100, 300 mg/kg wt). Lipopolysaccharide (Calbiochem, Germany) was dissolved in 0.9% saline, after which the mice were given a single intranasal LPS challenge (0.5 mg/kg body wt). The negative control group was administered only saline, while mice in the positive control group were administered dexametasone (10 mg/kg body wt). On day 3 after LPS stimulation, each group of mice was sacrificed. The long-term effects of PM014 on lung damage were assessed using an elastase- and LPS-induced animal model [16]. Animals were exposed to 1.2 units of porcine pancreatic elastase (PPE; Sigma Aldrich, USA) by the intranasal route on day 1 and to 7 *μ*g of LPS on day 4 of the week for four consecutive weeks. One week after the last exposure to LPS, each group of mice was sacrificed for further studies. Each group of mice was administered PM014 via an oral injection 2 hr prior to PPE or LPS stimulation (Figure 6(a)). The negative control group was administered only saline, while mice in the positive control group were administered dexametasone intraperitoneally (10 mg/kg body wt).

### 2.5. Analysis of Lung Inflammatory Cells

Phosphate-buffered saline (PBS) was slowly infused into the lungs and then withdrawn via a cannula that had been inserted into the trachea. The number of total and differential cells in the bronchoalveolar lavage (BAL) fluid were then determined using a hemocytometer. In addition, differential cell counts were conducted on slides that were prepared by cytocentrifugation and Diff-Quick staining. Approximately 500 cells were counted per slide. The BAL fluids were then centrifuged, after which the supernatants were stored at −80°C until needed.

### 2.6. Measurements of IL-6 and TNF-*α* in BAL Fluid

The levels of IL-6 and TNF-*α* in the BAL fluid were determined using a commercial enzyme immunoassay kit (BD Pharmingen, USA) according to the manufacturer's protocols. The detection limits for the IL-6 and TNF-*α* ELISAs were 7.8 pg/mL.

### 2.7. Preparation of Lung Tissues and Histology and Immunohistochemistry

Lung tissues were fixed in 4% paraformaldehyde solution and then embedded in paraffin. For histological examination, 4 *μ*m sections of lung tissue were stained sequentially with hematoxylin and eosin (H&E) or periodic acid Schiff [17] (Sigma, USA). For the immunohistochemistry of neutrophil elastase (NE) and proliferating cell nuclear antigen (PCNA), 4 *μ*m sections of lung tissue were treated with 0.3% H_2_O_2_-methanol for 30 min to block the endogenous peroxidase, after which they were incubated at 4°C overnight with anti-NE goat polyclonal antibody (1 : 50 dilution; Santa Cruz Biotechnology, USA) or anti-PCNA rabbit polyclonal antibody (1 : 50 dilution; Santa Cruz Biotechnology, USA). The slides were then incubated with avidin-biotin peroxidase complex (ABC kit, Vector Laboratories, USA), after which the color was developed with 3,3′-diaminobenzidine tetrachloride (DAB, Vector Laboratories, USA). Following immunohistochemical staining, the slides were counterstained with the Harris hematoxylin for 2 min and then mounted with Canada balsam (Showa Chemical Co. Ltd., Japan).

### 2.8. Statistical Analysis

Data are presented as the means  ±  SEM. Analysis of data was conducted using the GraphPad Prism software (Version 4, USA). The differences between study groups were determined by one-way ANOVA and Newman-Keuls' multiple comparison test. A *P* < 0.05 was considered to be statistically significant. 

## 3. Results

### 3.1. DART-MS Analysis of PM104

**Figure 1:**
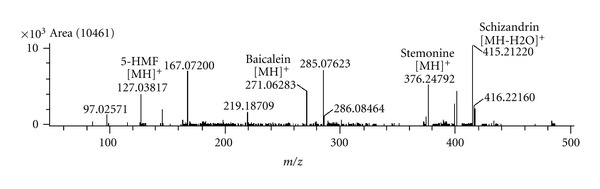
A representative DART-MS spectrum of PM104 extract.

Although the identity and quality of medicinal herbs used in this study was guaranteed by Sun Ten Pharmaceutical Co., DART-MS measurement were conducted on the PM104 preparation to reconfirm the composition of the herbal medicines. A representative DART-MS spectrum of PM104 is shown in Figure 1. Elemental compositions of major peaks were calculated using built-in software based on the exact mass numbers of the elements. The mass-to-charge ratios of 127.038 and 376.248 were identified as protonated 5-HMF and stemonine, which are the chemical markers of *Rehmannia glutinosa* and *Stemona sessilifolia*, respectively [18, 19]. The protonated ions of m/z 271.061 were identical to the molecular weight of baicalein from Scutellaria baicalensis. Since the sugar moiety of baicalin is often cleaved during DART ionization, this ion peak can be attributed to the presence of baicalin in the sample. The ion peak at m/z 415.212 can be identified as the dehydrated form of schizandrin ion, which was detected from *Schizandra chinensis* by DART-MS analysis [20]. Some chemical markers such as asparagine of *Asparagus cochinchinensis*, amygdalin from *Prunus armeniaca*, and paeoniflorin from *Paeonia suffruticosa* could not be detected from the crude extract of PM014. Although all chemical markers of seven medicinal herbs of PM014 could not be identified by DART-MS analysis, the identities of the four major components of *Rehmannia glutinosa*, *Stemona sessilifolia*, *Scutellaria baicalensis*, and *Schizandra chinensis* were successfully confirmed and the DART-MS fingerprint of PM014 could be used to determine the identity of the sample used in all experiments. The empirical mass numbers of the identified compounds differed from their theoretical mass numbers by less than 2 mmu. The high resolution power of the reflectron TOF-MS enabled efficient confirmation of the detected molecular ions by comparison of the measured molecular mass with the corresponding theoretical molecular mass.

### 3.2. The Therapeutic Effect of Mixed Herbs, PM014, and Individual Herbs

**Figure 2:**
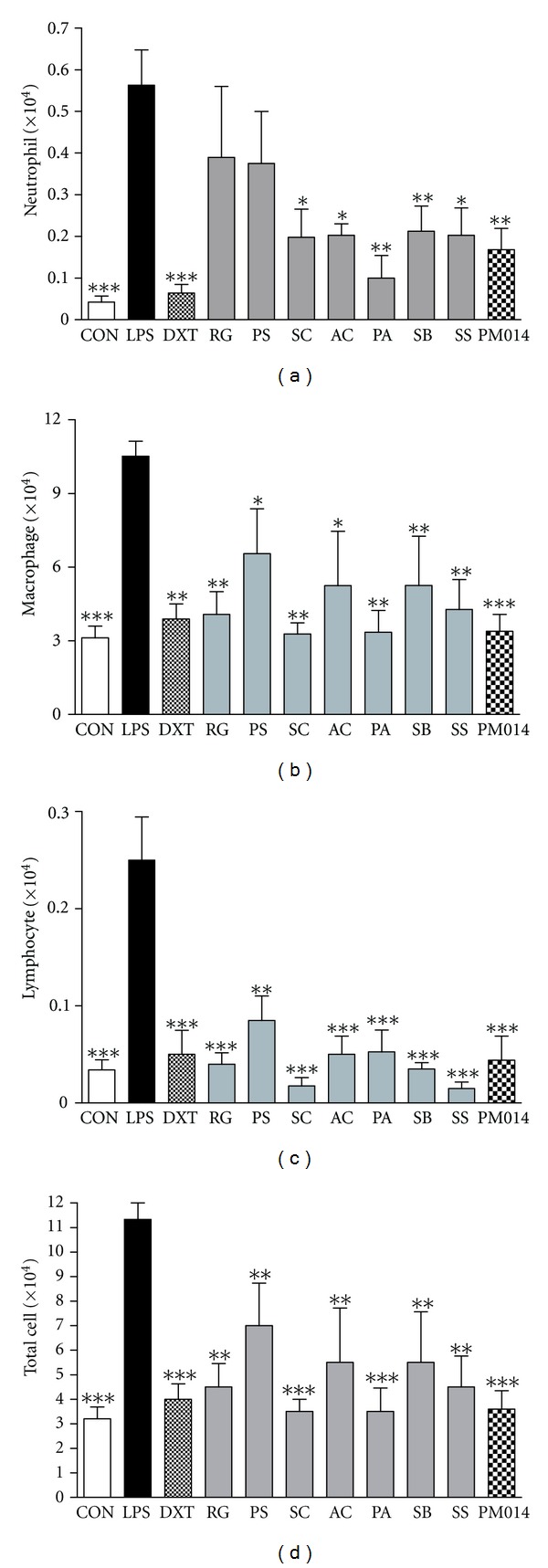
The therapeutic effect of mixed herbs, PM014, and individual herbs. Lipopolysaccaride was administrated to mice to induce a disease that resembles COPD. The numbers of neutrophils, macrophages, lymphocytes, and total cells were determined in BAL fluid. Con: control, LPS: lipopolysaccaride, DXT: dexametasone, RG: *Rehmannia glutinosa*, PS: *Paeonia suffruticosa*, SC: *Schizandra chinensis,* AC: *Asparagus cochinchinensis*, PA: *Prunus armeniaca,* SB: *Scutellaria baicalensis*, SS: *Stemona sessilifolia.* Data are expressed as the mean number of cells ± S.E.M. (*P < 0.05, **P < 0.01, ***P < 0.001 versus LPS; *n* = 4–6).

At 3 days after LPS challenge, a significant increase in the total number of cells was observed in the LPS-treated group when compared with the saline-treated control group. In addition, the influx of macrophages, neutrophils, and lymphocytes was remarkably higher in the LPS-treated group than the control group. Individual herb extracts in PM014 also showed an attenuation effect on the immune cells influx; however, treatment with herbal mixture, PM014, exerted less recruitment of all immune cells toward lungs than individual herbal treatment (Figure 2). 

### 3.3. Effect of PM014 on Acute Pulmonary Inflammation

**Figure 3:**
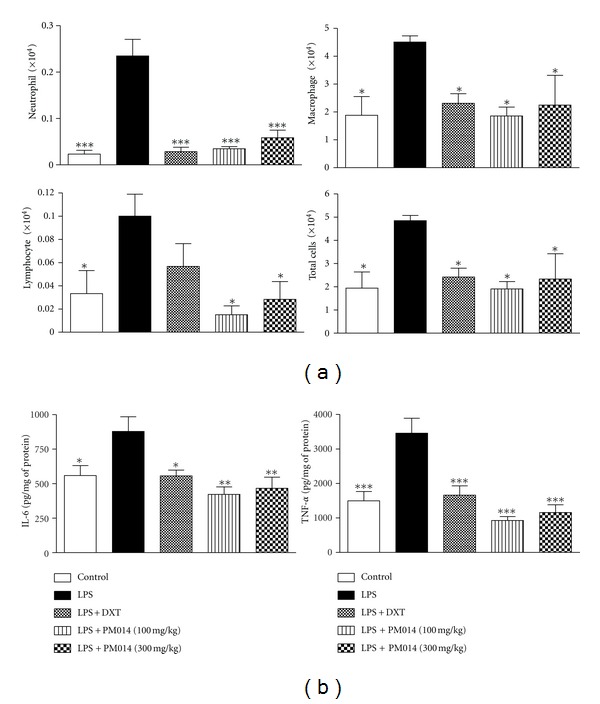
Effect of PM014 on immune cell profiles and pro-inflammatory cytokines. (a) Effect of PM014 on immune cell profiles in BAL fluid. The numbers of neutrophils, macrophages, lymphocytes, and total cells were determined in BAL fluid. (b) The levels of IL-6 and TNF-*α* in BAL fluid were determined by ELISA. Data are expressed as the mean number of cells ± SEM (*P < 0.05, **P < 0.01, ***P < 0.001 versus LPS; *n* = 6).

At 3 days after LPS challenge, a significant increase in total cells was observed in the LPS group when compared with the PM014 pretreatment group. In addition, the influx of macrophages, neutrophils, and lymphocytes in the LPS group was remarkably higher than that in the PM014 pretreatment group (Figure 3(a)). TNF-alpha and IL-6 are known to be pro-inflammatory cytokines that contribute to LPS-induced lung inflammation. Therefore, the level of these cytokines in BAL fluid at 3 days after LPS challenge was measured. Treatment with PM014 2 h prior to the administration of LPS significantly reduced the levels of TNF-*α* as effectively as dexamethasone. The production of IL-6 was also significantly decreased in the PM014 pretreatment group when compared to the LPS group (Figure 3(b)).

### 3.4. Histological Evaluation of the Lungs

**Figure 4:**
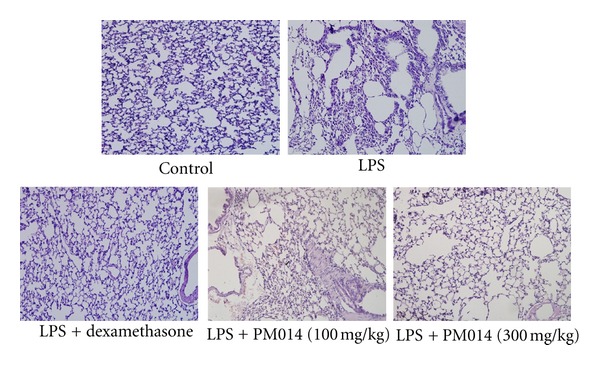
Effect of PM014 on lung tissue. LPS treatment induced parenchymal and airway inflammation. Mouse lung sections were stained with hematoxylin and eosin (magnification ×200).

To determine if PM014 exerted an effect on LPS-induced lung damage, we stained lung sections with hematoxylin and eosin (H&E). LPS-challenged mice showed a heavy infiltration of inflammatory cells and exudative changes in the peribronchial layers and intraluminal areas of the large and small bronchi. However, treatment with PM014 ameliorated this lung inflammation (Figure 4). 

### 3.5. Immunohistochemical Findings of PM014

**Figure 5:**
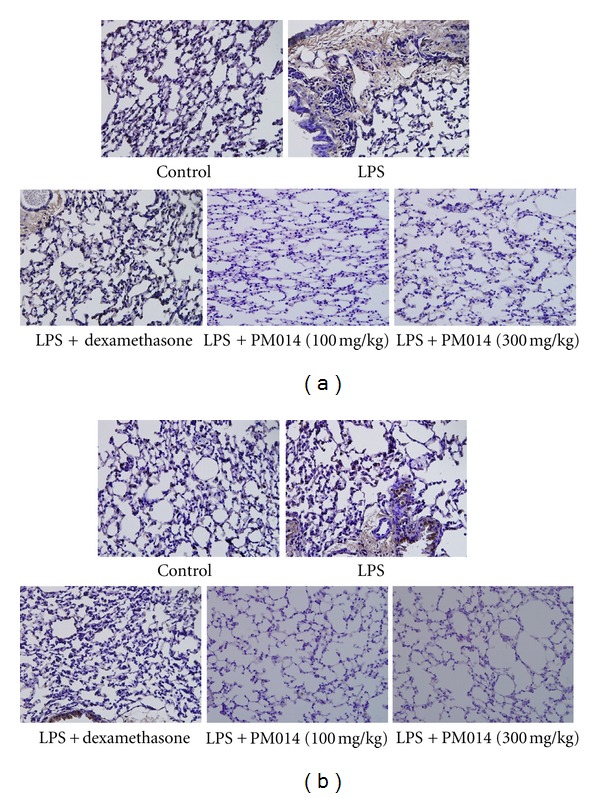
Effect of PM014 on the expression of neutrophil elastase and proliferating cell nuclear antigen. Mouse lung sections were stained immunohistochemically. Mouse lung sections were stained with anti-NE goat polyclonal antibody (a) or with anti-PCNA goat polyclonal antibody (b). After the slides were incubated with avidin-biotin peroxidase complex, the color was developed with 3,3′-diaminobenzidine tetrachloride.

To evaluate the effects of PM014 on LPS-induced lung damage, immunohistochemical staining for NE and PCNA was conducted. NE was strongly expressed in the LPS group, while the levels of NE were notably reduced in the PM014 pretreatment group (Figure 5(a)). Concomitant with NE expression, PCNA was also highly expressed in the LPS group when compared to the PM014 pretreated group (Figure 5(b)).

### 3.6. Effect of PM014 on the Repeated PPE and LPS Exposure Disease Model

**Figure 6:**
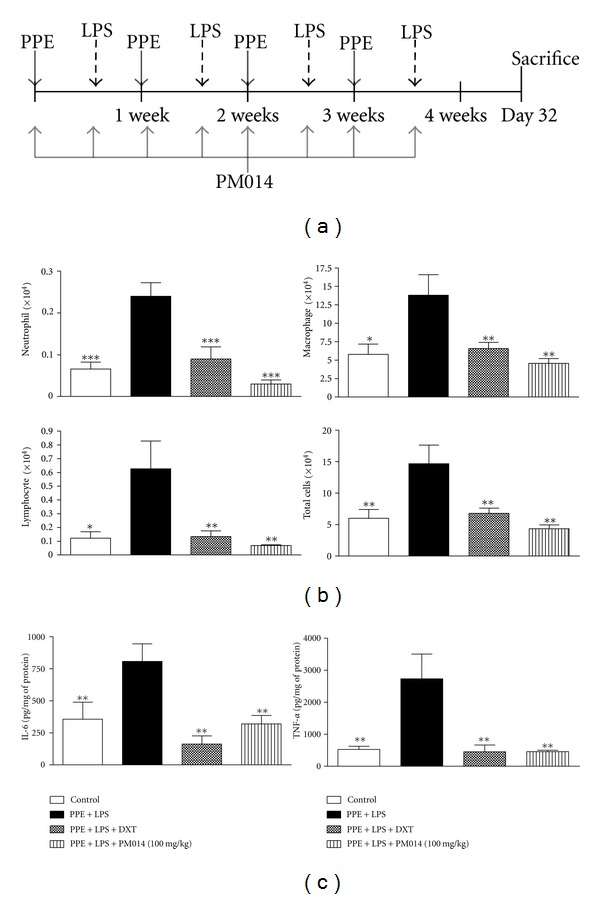
Effect of PM014 on lung inflammation in elastase/LPS-exposed mice. (a) Experimental plan of repeated elastase and LPS exposure. (b) Effect of PM014 on immune cell profiles in BAL fluid. The numbers of neutrophils, macrophages, lymphocytes, and total cells were determined in BAL fluid. (c) The levels of IL-6 and TNF-*α* in BAL fluid were determined by ELISA. PPE: porcine pancreatic elastase, LPS: lipopolysaccharide. Data are expressed as the mean number of cells ± SEM (*P < 0.05, **P < 0.01 versus LPS; *n* = 5-6).

**Figure 7:**
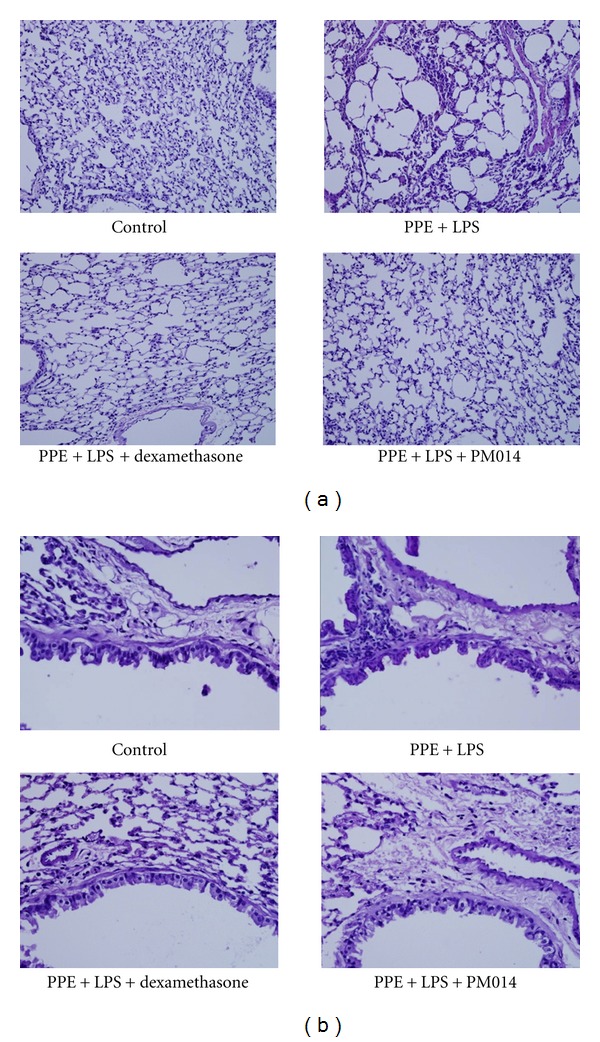
Effect of PM014 on lung tissue changes in elastase/LPS-exposed mice. (a) Mouse lung sections were stained with hematoxylin and eosin (magnification ×200). (b) Mouse lung sections were stained with PAS (magnification ×400). PPE: porcine pancreatic elastase, LPS: lipopolysaccaride.

To potentiate the therapeutic effect of PM014 in experimental animals, long-term exposure of mice to LPS for four weeks was conducted. In addition, we augmented the LPS model by treatment with elastase, which is a well-known inducer of emphysema in lung tissue [21]. Seven days after final treatment with LPS, mice were sacrificed for further studies. The influx of immune cells was remarkably higher than that in the PM014 pretreatment group (Figure 6(b)). Treatment with PM014 significantly reduced the levels of TNF-*α* and IL-6 when compared to the PPE/LPS group (Figure 6(c)). Mice treated with PPE/LPS showed alveolar destruction, which resulted in enlarged air spaces, indicating emphysematous change. In contrast, mice treated with PM014 showed less tissue damage (Figure 7(a)). Consistent with previous results, PPE/LPS-treated mice showed more PAS-positive cells in the large airways. However, the increase in PAS-positive goblet cells in the PM014 group was significantly lower than that observed in the PPE/LPS group (Figure 7(b)). Taken together, these findings indicate that treatment with PM014 had a powerful therapeutic effect on the long-term lung inflammation model.

## 4. Discussion

In this study, infiltration of inflammatory cells in BAL fluid and in the lung parenchyma was observed following exposure to LPS. Concomitant with the influx of neutrophils, increased levels of the proinflammatory cytokines TNF-*α* and IL-6 were observed in the BAL fluid of LPS-exposed mice. In addition, the expression of NE and PCNA in the airway was found to increase following LPS exposure. Notably, treatment of PM014 attenuated the hazard effect of LPS.

Herbal mixtures are widely used as traditional medicines to treat many different types of disease [22, 23]. In Korean traditional medicine, herbs are generally used as a mixture. Therefore, we initially compared the effects of each herb in PM014 and the herbal mixture PM014. LPS is a major proinflammatory component of gram-negative bacteria that is present in cigarette smoke and evokes pulmonary and systemic inflammation in humans [24]. In the present study, infiltration of inflammatory cells in BAL fluid was observed after the first exposure to LPS. Exposure to LPS induced several pathological changes such as inflammatory cell accumulation in the lung parenchyma, hyperplasia of goblet cells, hypersecretion of mucus, enlargement of alveoli, and increased collagenic and elastic structures in the alveolus in this study. The pathological changes observed in this study were very similar to the clinical features of COPD patients [25]. In this LPS exposure model, herbal mixture PM014 showed constant efficacy when compared to individual herb extract. 

Interestingly, PM014 pretreatment ameliorated the lung pathological changes in short-term and long-term LPS exposure models. These results may suggest that PM014 is a powerful anti-inflammatory agent that reduces the acute and chronic accumulation of inflammatory cells induced by LPS. In this study, TNF-*α* and IL-6 were also increased in the BAL fluid of LPS-treated mice, while the levels of these cytokines were significantly lower in the BAL fluid of PM014-treated mice. TNF-*α*, an early proinflammatory cytokine, is believed to trigger the activation of other proinflammatory cytokines such as IL-6 and IL-8 [26]. TNF-*α* also activates nuclear factor-*κ*B, which increases IL-8 gene transcription, thereby inducing the release of IL-8 from the airway epithelium and neutrophils. IL-8, a CXC chemokine, is a neutrophil chemoattractant and activator [25]. In our study, PM014 downregulated pro-inflammatory cytokine production in both acute and chronic lung inflammation. Therefore, treatment with PM014 may act on downstream events, including the influx of inflammatory cells and the levels of mediators aggravating the inflammatory response.

In the present study, NE expression and PCNA levels were remarkably reduced by treatment with PM014. NE from neutrophils has been linked to a variety of inflammatory diseases, including COPD and cystic fibrosis. In addition, the results of several studies have suggested that NE plays a key role in the pathogenesis of both emphysema and lung fibrosis during the inflammatory process [27]. Together with NE and PCNA analysis, the long-term PPE/LPS exposure histopathological data showed that PM014 administration inhibited the progression of emphysema and goblet cell metaplasia. These data demonstrated that PM014 could be sufficient to prevent structural changes typical of COPD including pulmonary emphysema, diffuse lung inflammation, and goblet cell metaplasia.

## 5. Conclusions 

This study provides evidence that treatment with PM014 exerts preventive and therapeutic effects against COPD-like animal models in mice. The remarkable effect exerted by PM014 suggests that it has the potential for use in the treatment of lung inflammation. Therefore, further study to elucidate the mechanisms by which the effects of PM014 occur should be conducted to aid in the discovery of new therapeutic agents for the lung inflammatory disease such as COPD.

## Figures and Tables

**Table 1:** Formula of PM014 and chemical markers of each medicinal herb.

Formula of PM014	Weight	Chemical marker
Root of *Stemona sessilifolia * (Bai Bu)	4 g	Stemonine
Root of *Asparagus cochinchinensis * (Tian Men Dong)	8 g	Asparagine
Root of *Scutellaria baicalensis * (Huang Qin)	6 g	Baicalin
Fruit of *Schizandra chinensis * (Wu Wei Zi)	8 g	Schizandrin
Root of *Rehmannia glutinosa * (Shu Di Huang)	16 g	5-HMF
Seed of *Prunus armeniaca* (Xingren)	6 g	Amygdalin
Cortex of *Paeonia suffruticosa* (Mudanpi)	8 g	Paeoniflorin

Total	56 g	
